# Supplementary material for: Water Column Microbial Communities Vary along Salinity Gradients in the Florida Coastal Everglades Wetlands
Source: Microorganisms. 2022 Jan 20;10(2):215. doi: 10.3390/microorganisms10020215 (PMC8874701; doi:10.3390/microorganisms10020215)
Supplement: Supplementary file 1 [file microorganisms-10-00215-s001.zip › microorganisms-1534040-supplementary.pdf]

Supplementary Materials

Table S1. Quantitative effects of environmental parameters, ecosystem type (freshwater marshes, ecotone, marine) and transect (SRS, TS/Ph, FB+) on the bacterial community variation by permutational multivariate analysis of variance (using 'adonis' function of vegan package) based on weighted UniFrac distance. R^2 values present the proportion of variation constrained by factors.

	**F model**	**R^2**	**P value**
ecosystem type	6.6624	0.44593	0.001**
transect	2.0339	0.06807	0.085
Chl a	2.2035	0.07374	0.064
Salinity	3.0081	0.10067	0.019*
TP	1.5980	0.05348	0.169
TN	1.5388	0.05150	0.171
BA	1.4587	0.04882	0.197
BP	0.7153	0.02394	0.677

+FB was treated separately as there was no marine counterpart in the SRS transect, *significant, **highly significant.

Table S2. Quantitative effects of environmental parameters, ecosystem type (freshwater marshes, ecotone, marine) and transect (SRS, TS/Ph, +FB) on the eukaryotic microbial community variation by permutational multivariate analysis of variance (using 'adonis' function of vegan package) based on weighted UniFrac distance. R^2 values present the proportion of variation constrained by factors.

	**F model**	**R^2**	**P value**
ecosystem type	2.56750	0.26611	0.002**
transect	1.37241	0.07112	0.141
Chl a	1.78744	0.09263	0.050*
Salinity	2.10254	0.10896	0.010**
TP	0.76891	0.03985	0.757
TN	1.32605	0.06872	0.197
BA	1.32391	0.06861	0.197
BP	1.48022	0.07671	0.120

+FB was treated separately as there was no marine counterpart in the SRS transect, *significant, **highly significant.
